# Infection Prevention and Control: Practical and Educational Advances

**DOI:** 10.3390/tropicalmed7080148

**Published:** 2022-07-26

**Authors:** Constantinos Tsioutis, Spyridon A. Karageorgos

**Affiliations:** 1 School of Medicine, European University Cyprus, Egkomi 2404, Cyprus; spiroskarageorgo@gmail.com; 2 First Department of Pediatrics, Aghia Sophia Children’s Hospital, National and Kapodistrian University of Athens, 11527 Athens, Greece

Infection prevention and control (IPC) is associated with improved healthcare, better quality of life and cost-effectiveness in disease prevention. On the other hand, limited or inadequate application of IPC is associated with high disease burden and poor health outcomes. This is why IPC is now considered an emerging global priority by the World Health Organization, which outlines important priorities for the improvement of IPC practice and research on a worldwide basis [1].

The practice of IPC is not limited to specific healthcare sectors or geographic regions; IPC spans across the community, healthcare settings, hospitals, and involves different activities, including policy drafting and application, education, epidemiological surveillance, diagnostics, practical measures in healthcare provision, and treatment. Application of IPC is a dynamic process, directly associated to research and surveillance. The success of IPC is relevant to the degree of adjustment to local culture, needs and priorities, while different settings and different diseases require different measures.

The ongoing topical collection “Infection Prevention and Control: Practical and Educational Advances” under the section “Infectious Diseases” in Tropical Medicine and Infectious Diseases (ISSN 2414-6366) aims to highlight the importance and heterogeneity in IPC research and practice worldwide. The collection started in May 2020 and currently, 25 manuscripts from across the globe have been published. As of 14 July 2022, the collection achieved 49,725 views and 60 citations. This editorial presents a brief overview of the scientific content of this topical collection.

The key areas covered in this topical collection are as follows: (1) diagnostics and screening, (2) epidemiologically important diseases, (3) antimicrobial-resistant bacteria, (4) an outbreak investigation, (5) updates on policies, practical issues and measures, and (6) issues surrounding COVID-19. Among the published manuscripts, 17 were based on primary research and 8 were either reviews or systematic reviews. Primary research studies were carried out in Europe (eight studies), Africa (five studies), Asia (two studies), North America (one study), and South America (one study). Another point of interest is the variety of pathogens discussed, including tuberculosis, Chikungunya virus, hantaviruses, multidrug resistant bacteria, *Yersinia* infections, hepatitis C, schistosomiasis, rabies, and others.

## Diagnostics and Screening

The quality of latent tuberculosis infection screening using an interferon-gamma release assay was assessed in a tertiary center in Ireland [2]. After evaluating its use in 1507 patients between 2016 and 2018, several issues were spotted regarding scaling of the screening process and treatment initiation. In a study by Gunnink et al., the national screening protocols for multidrug-resistant microorganisms, as well as local screening protocols between two hospitals on the Dutch-German border, were compared [3]. Practicality of the screening protocols was sufficient, but the comparison detected several issues that may impair consistency between different protocols and complicate patient care. Osa et al., evaluated the performance of MALDI–TOF MS compared to conventional methods (VITEK2 or manual biochemical methods) in the Philippines at the species and genus level among bacterial and fungal isolates [4]. A total of 3530 isolates were tested; concordance rates were high (in total, 96.2% for species and 99.9% for genus level). Concordance with VITEK2 was 95.8% (species level) and 99.8% (genus level), and 96.6% (species level) and 99.9% (genus level), compared with manual biochemical testing.

## Epidemiologically Important Diseases

Hemachudha et al., presented the causes of encephalitis in Thailand among 1700 cases from 2013 to 2018, tested with multiplex PCR for different pathogens [5]. The most common isolates were EBV (27%), enterovirus (17.3%), VZV (11.3%), (9.7%), HSV-1 (7.8%), and HHV-6 (6.3%), indicating the increasing prevalence of enteroviral encephalitis compared to previous years. Vilibic-Cavlek et al., provided a detailed review of lymphocytic choriomeningitis virus (LCMV), a neglected rodent-borne zoonotic virus with worldwide distribution, which although rare, is associated with significant burden of disease, mortality and long-term sequelae especially in newborns, transplant patients and pregnant women [6]. Aula et al., extensively reviewed schistosomiasis in Africa [7], the evolution of rabies in South America during 2009–2018 was analyzed by compiling data from different sources by Meske et al. [8], whereas Ioannou et al., described 12 patients with *Yersinia* endocarditis in a systematic review of the literature [9]. Hepatitis C (HCV) remains a significant issue in Eastern Libya according to the study by Ismail et al., where among 612 individuals who tested positive for HCV antibodies in Tobruk Medical Center from 2005 to 2020, no follow-up RNA test was detected and no links to outpatient care and treatment were found [10]. Riccò et al., detected significant gaps in knowledge and risk perceptions among 223 Italian physicians (42% were occupational physicians) in relation to hantaviruses in at-risk workers [11], whereas Laycock et al., reviewed the literature on tuberculosis among adolescents and young adults (aged 10–24 years) and identified areas for further research and interventions to prevent and treat tuberculosis in this age group [12]. Cardiac symptoms are not an uncommon manifestation in Chikungunya infection and may have high mortality rates, as evidenced by an extensive literature review by Traverse et al. [13]. Finally, two studies reported interesting cases and reviewed the literature on severe odontogenic infections during pregnancy [14] and Campylobacter myocarditis [15].

## Antimicrobial-Resistant Bacteria

A systematic review by Lena et al., found 23 studies that reported on the presence of methicillin-resistant *Staphylococcus aureus* (MRSA) between 2000 and 2020 on healthcare workers’ clothing [16]. Despite variations in study methodologies, a high rate of MRSA presence was reported, particularly in long-sleeved white coats and ties, as well as evidence of inappopriate laundering techniques. Onduru et al., found a rate of 16.7% of ESBL-producing Enterobacterales in community samples in Blantyre, Malawi, as well as a 4% resistance rate to meropenem [17]. Among 137 residents in 6 nursing homes in Crete, Greece, Moschou et al., reported rectal colonization by multidrug-resistant microorganisms in 18% of 255 isolates, primarily extended-spectrum-lactamase (ESBL)-producing organisms (26%) [18]. Antimicrobial use within 3 months was found to be associated with higher carriage. Spernovasilis et al., investigated the perceptions, attitudes and practices of 202 hospital doctors regarding the management of multidrug-resistant organisms after the implementation of an antimicrobial stewardship programme (ASP) in Greece, who demonstrated positive attitudes towards the effectiveness of the ASP in their daily practice and favored the prospective audit and feedback strategy compared to preauthorization [19].

## Outbreak Investigation

A cluster of eight *Plasmodium ovale* infections among Belgian military personnel was recorded after deployment in the Democratic Republic of Congo, with a median delay between return from deployment and diagnosis of 103 (IQR 62-339) days, indicating that non-specific symptoms and lack of sensitive diagnostic tools in the field might have led to underestimation of infection [20].

## Policies, Practical Issues and Measures 

Tourapi and Tsioutis suggested the Circular Policy approach to the management of vector and vector-borne diseases, a holistic policy for national and local strategies to be effective and adaptable in line with the global vector control response [21]. Malloy et al., investigated the cost-effectiveness of interventions during the 2017 *Yersinia pestis* (plague) outbreak in Madagascar, which caused 2417 cases and 209 deaths [22]. Expanded access to antibiotic treatment with doxycycline was the most cost-effective intervention, highlighting the importance of widespread and rapid access to antibiotics upon recognition of the outbreak. A perioperative clinical practice protocol for prevention of periprosthtetic joint infections (PJI) in high-risk patients, based on an extensive literature review, was developed by an international panel formed by the European Knee Associates [23]. Various preoperative, intraoperative and postoperative modifiable factors that affect PJI risk were recognized.

In an interesting study where 165 orthopedic operations were surveyed with 3 or more observers present, in addition to the surgical team in Spain, the presence of observers was not associated with a higher rate of complications compared to the literature [24]. A cross-sectional study using the WHO hand hygiene self-assessment framework in two hospitals in Sierra Leone during the COVID-19 pandemic recorded several areas for improvement, as recorded hand hygiene compliance was as low as 17–20.8% [25].

## COVID-19

Kalligeros et al., showed early on during the first months of COVID-19 vaccination in the USA that even partial vaccination (ie. single dose) was associated with a lower probability of death in hospitalized patients with COVID-19 [26].

## Conclusions

As the current topical collection continues to receive new submissions, the importance and heterogeneity of IPC as a broad medica field is further unfolded, thus highlighting its significance in improving health outcomes across the world and for different diseases. The field of IPC is still young and as research progresses, knowledge and competency expands. The COVID-19 pandemic has revealed the need for further research in IPC, whereas the enormous shift of healthcare provision towards COVID-19 for the past two years will undoubtedly leave its mark on the progress and dissemination of other infectious diseases [27,28]. Therefore, raising awareness and enhancing knowledge and IPC practice through continuous research and education will help improve quality of healthcare and patient outcomes worldwide.
